# Lactate production yield from engineered yeasts is dependent from the host background, the lactate dehydrogenase source and the lactate export

**DOI:** 10.1186/1475-2859-5-4

**Published:** 2006-01-30

**Authors:** Paola Branduardi, Michael Sauer, Luca De Gioia, Giuseppe Zampella, Minoska Valli, Diethard Mattanovich, Danilo Porro

**Affiliations:** 1 Università degli Studi di Milano – Bicocca, Dipartimento di Biotecnologie e Bioscienze, P.^zza ^della Scienza 2, 20126 Milano, Italy; 2 Institute of Applied Microbiology, BOKU – University of Natural Resources and Applied Life Sciences, Muthgasse 18, A-1190 Wien, Austria; 3 Fh-campus wien – University of Applied Sciences, School of Bioengineering, Muthgasse 18, A-1190 Wien, Austria

## Abstract

**Background:**

Metabolic pathway manipulation for improving the properties and the productivity of microorganisms is becoming a well established concept. For the production of important metabolites, but also for a better understanding of the fundamentals of cell biology, detailed studies are required. In this work we analysed the lactate production from metabolic engineered *Saccharomyces cerevisiae *cells expressing a heterologous lactate dehydrogenase (*LDH*) gene. The *LDH *gene expression in a budding yeast cell introduces a novel and alternative pathway for the NAD^+ ^regeneration, allowing a direct reduction of the intracellular pyruvate to lactate, leading to a simultaneous accumulation of lactate and ethanol.

**Results:**

Four different *S. cerevisiae *strains were transformed with six different wild type and one mutagenised *LDH *genes, in combination or not with the over-expression of a lactate transporter. The resulting yield values (grams of lactate produced per grams of glucose consumed) varied from as low as 0,0008 to as high as 0.52 g g^-1^. In this respect, and to the best of our knowledge, higher redirections of the glycolysis flux have never been obtained before without any disruption and/or limitation of the competing biochemical pathways.

**Conclusion:**

In the present work it is shown that the redirection of the pathway towards the lactate production can be strongly modulated by the genetic background of the host cell, by the source of the heterologous Ldh enzyme, by improving its biochemical properties as well as by modulating the export of lactate in the culture media.

## Background

Metabolic engineering can be defined as the directed improvement of product formation or cellular properties through the modification of specific biochemical reactions or introduction of new ones with the use of recombinant DNA technology. Aimed to produce single compounds, metabolic engineering necessarily includes the modification of the cellular pathway(s) as well as the redirection of the energy toward the production itself (see for a review [1]). The existing metabolic engineering applications are the result of more than two decades of global experience developing processes for the production of fine chemicals, vitamins, nutraceuticals and animal nutritional supplements such as amino acids (as examples, see [2-5]). Given their relatively low complexity, the first biotechnological applications have been developed in microorganisms.

Different research teams have been involved in the production of lactate from metabolic engineered yeasts such as *Saccharomyces cerevisiae *[6-13], *Kluyveromyces lactis *[14,15], *Torulaspora delbrueckii *[16] and *Zygosaccharomyces bailii *[17]. L-Lactic acid, first discovered by the Swedish chemist Scheele (1780), has been traditionally used as a food preservative and food flavoring compound [18]. Recently, it has received attention since it can be used to produce a biodegradable polymer with plastic properties. The market for this organic acid is rapidly growing, exceeding several hundred million dollars annually [10]. This carboxylic acid is currently mainly produced using lactic-acid bacteria, such as various *Lactobacillus *species,(via an, anaerobic fermentation that operates optimally at pH values where the salt of the organic acid rather than the free acid is formed, although free lactic acid is preferred for most industrial processes [18]. The use of microorganisms like yeasts that are more tolerant to low pH values than the current production organisms, could strongly decrease the amount of neutralizing agents required and lower the cost of down-stream processing. Pyruvate is the end product of glycolysis; it can be further metabolized either by the pyruvate dehydrogenase complex (Pdh, EC 1.2.4.1) to acetyl-coenzyme A or by pyruvate decarboxylase (Pdc, EC4.1.1.1) to acetaldehyde and subsequently to ethanol. In previous works it has been shown that the expression of a heterologous lactate dehydrogenase (Ldh, EC 1.1.1.27) gene in the above mentioned yeast hosts introduces a new and alternative pathway for the NAD^+ ^regeneration, allowing a direct reduction of the intracellular pyruvate to lactate leading to a simultaneous formation of ethanol and lactic acid [7]. Only following a partial or a full replacement of the ethanol production by the lactate production, obtained in recombinant yeast cells lacking the Pdc and/or Pdh activities, it has been possible to obtain lactate production with high yield (max reported yield: 0.85, i.e., gram of lactate produced per gram of glucose consumed) values [15]. Theoretically, 2 moles of lactate and 2 moles of ATP are formed per mole of glucose consumed. In the present work we show that in *S. cerevisiae *cells, the yield value can be strongly modulated by the genetic background of the host, by the source of the heterologous Ldh enzymes, by improving their biochemical properties as well as by modulating the export of lactate in the culture media. Following all these approaches we have been able to improve the yield from values as low as 0,0008 to values as high as 0.52 without any modulation of the Pdc and/or Pdh activities.

## Results and discussion

### Expression of different LDH(s) in different yeast hosts

Lactic acid has been already produced by metabolically engineered yeast hosts (see Introduction). To better understanding the modulation of lactate production from the background of wild type *S. cerevisiae *cells, we firstly tested lactate production from different *S. cerevisiae *yeast strains transformed with the same integrative plasmid, pB1. As a model *LDH *gene we chose the mammalian Ldh-A lactate dehydrogenase. The transcription of the heterologous gene is under the control of the strong constitutive *S. cerevisiae *TPI (Triose Phosphate Isomerase) promoter. The resulting expression vector has been used to transform five different *S. cerevisiae *host strains. Transformed strains have been grown in batch shake-flask culture on 2% wv^-1 ^glucose-YNB based media. Further, to better understand the modulation of the production from different Ldh(s), we also tested the lactate production from the same *S. cerevisiae *yeast strain (GRF18U) transformed with the same integrative expression vector, but bearing different Ldh(s).

**Figure 1:**
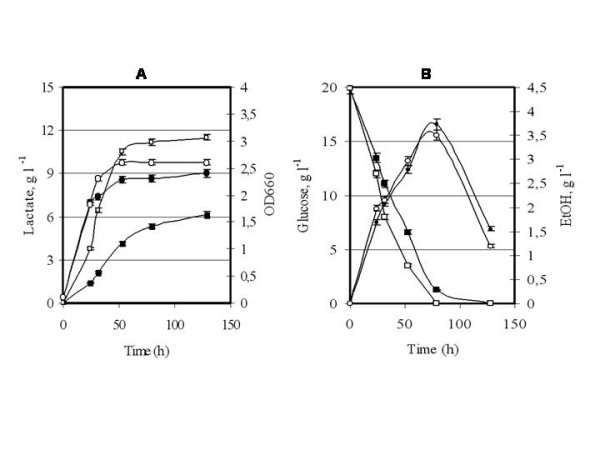
**Wild type Vs mutated Ldh**. Recombinant *S. cerevisiae *GRF18U cells expressing the wild type (closed symbols) or mutated (open symbols) *L. plantarum LDH *genes were shake-flask grown in glucose 2% wv^-1 ^minimal selective medium till stationary phase. Samples were collected at indicated times for optical density (OD 660 nm, circles; panel A), lactate (g l^-1^, squares; panel A), ethanol (g l^-1^, circles; panel B) and residual glucose (g l^-1^, squares; panel B) determinations.

Table 2 compares the lactate and the ethanol productions obtained. Independently from the yeast strain used and of the heterologous enzyme, the highest accumulation of lactate and ethanol were observed at the beginning of the stationary phase (reached, in these growth conditions, after about 30 hours from the inoculum), in coincidence with the exhaustion of glucose (data not shown; see for example also figure 1A and 1B). Findings reported indicate that the lactate production is strongly dependent from the background of the hosts. Furthermore, a higher lactate production comes along with a lower ethanol accumulation (data not shown). Productions ranging from 20 to 801 mg l^-1 ^have been obtained from different yeast hosts expressing one copy of the same bovine Ldh and yielding similar specific activities (ranging from 0,4 to 0,6 U mg^-1^). If two or three gene copies are expressed, the product levels (up to 1110 mg l^-1^) and activity (up to 0,8 U/mg) increase, even if the improvement not proportional to the gene copy number. Data reported in the Table 2 further indicate that different Ldh(s) lead to different lactate productions (from 20 to 6150 mg l^-1^). On one side it is interesting to underline that, despite the high Km value for pyruvate, high productions (4160 mg l^-1^) have been observed transforming the GRF18U yeast host with the *Lactobacillus casei *Ldh gene. On the other side, lactate concentrations of the same order (6150 mg l^-1^) have been obtained using the fructose1-6BP independent *L. plantarum *Ldh, which has a much lower Km value (Table 2).

**Table 1:** Plasmids (all integrative) utilized in this study*

Vector	Promoter	Heterologous protein	Selection marker	Reference
pB1	*Sc*TPI	*Bt*LDH	*URA*3	this study
pB2	*Sc*TPI	*Bt*LDH	*HIS*3	this study
pB3	*Sc*TPI	*Bt*LDH	*LEU*2	this study
pBME2	*Sc*TPI	*Bm*LDH	*URA*3	this study
pBST2	*Sc*TPI	*Bs*LDH	*URA*3	this study
pLC5	*Sc*TPI	*Lc*LDH	*URA*3	Brambilla *et al.*, 1999
pLC7	*Sc*TPI	*Lc*LDH	*HIS*3	Brambilla *et al.*, 1999
p022TLP	*Sc*TPI	*Lp*LDH	*HIS*3	this study
p012TLP	*Sc*TPI	*Lp*LDH	*URA*3	this study
p022TLPD94G	*Sc*TPI	*Lp*LDHD94G	*HIS*3	this study
p012TLPD94G	*Sc*TPI	*Lp*LDHD94G	*URA*3	this study
p012Jen1	*Sc*TPI	*Sc*Jen1	*URA*3	this study
p022Jen1	*Sc*TPI	*Sc*Jen1	*HIS*3	this study

*a complete description of plasmids construction is given in *Materials and Methods*

Abbreviations: *Sc*: *S. cerevisiae*; *Bt*: *Bos taurus*; *Bm*: *Bacillus megaterium Bs*: *Bacillus stearothermophylus*; *Lc*: *Lactobacillus casei*; *Lp*: *Lactobacillus plantarum*

**Table 2:** Lactate and ethanol productions from different *S. cerevisiae *hosts transformed with different Ldh(s).

	**plasmid**	***LDH***	**Lactate**	**EtOH**	**Yield**	**LDH activity**	**LDH Km**
**Strains**		(source)	mg l^-1^	mg l^-1^	g g^-1^	U mg^-1^	mM

W303-1A	pB1	*B. taurus*	20	6430	0,0010	0,4	1,00 [19]
CEN.PK	pB1	*B. taurus*	140	6340	0,0070	0,5	1,00
GRF18U	pB1	*B. taurus*	801	5750	0,0401	0,6	1,00
MB11	pLC5	*L. casei*	200	6110	0,0100	Nd	10,00 [19]
GRF18U	pB1; pB2	*B. taurus*	950	5730	0,0475	0,7	1,00
GRF18U	pB1; pB2; pB3	*B. taurus*	1100	5648	0,0550	0,8	1,00
GRF18U	pBST2	*B. stearotherm*.	128	6310	0,0064	0,6	0,03 [19]
GRF18U	pBME2	*B. megaterium*	1371	5672	0,0686	0,7	Nd
GRF18U	pLC5	*L. casei*	4160	4270	0,2080	5,5	10,00
GRF18U	p022TLP	*L. plantarum*	6150	3730	0,3075	3,2	1,50 (this study)

All the indicated recombinant yeast strains, together with the respective control here not reported, have been grown in shake-flasks on 2% (w/v) glucose-YNB based media till stationary phase, reached after about 30 hours after the inoculum. Cultures were independently repeated, after the screening of independent transformants, (at least) three times, rising to comparable results. In the table one fermentation per each set of experiment is reported, being the independent kinetics reproducible among themselves.

Lactate and ethanol production (mg l^-1^, columns 4 and 5, respectively) correspond to the highest measured production levels.

The Yield value (column 6) represents the grams of lactate produced per gram of glucose consumed (max theoretical yield = 1).

Ldh activity (column 7) has been determined at the highest lactate production level.

Km values against NADH are comprised between 0.001 and 0.0045 mM [19].

Km apparent value of mitocondrial pyruvate transporter is 0,3 mM [44]

Nd: not determined.

In general, and as expected, it can be concluded that the higher the specific activity is, the more efficient is the conversion of pyruvate to lactate.

Similar results have been obtained transforming the host cells with identical centromeric plasmids bearing different Ldh(s) under the control of the same *Sc*TPI promoter (data not shown).

### Improving lactate production and yield by protein engineering

The Ldh enzyme is composed of four identical subunits [19]. All the bacterial Ldh enzymes listed in Table 2, with the exception of the *L. plantarum *Ldh, require fructose 1-6BP as a cofactor. It has been proposed that the rate limiting step of the enzymatic reaction corresponds to the conformational rearrangement of the so-called catalytic loop [20]. In particular, the movement of the catalytic loop is necessary to allow pyruvate binding and L-lactate release.

Looking at a computational development of a better Ldh enzyme, we initially took into consideration the spiny dogfish (*Squalus acanthias*) Ldh protein. This enzyme was selected because the spatial structure of the enzyme, which is required for the docking simulations, is available in literature [21]. Because of the high sequence similarity with Ldh proteins from other sources, simulations data can be extended to the other enzymes of this family. The comparison between the X-ray structures of the apo and holo form of Ldh from *S. acanthias *reveals that the aminoacid Asn108 could play a relevant role in the dynamic behaviour of the catalytic loop. The effect of Asn108 replacement on the potential energy profile related to the conformational rearrangement of the catalytic loop has been evaluated *in silico*, according to the procedure outlined in Methods. In particular, the computational investigation of five Ldh mutants in which Asn108 has been replaced by Asp, Ala, Gly, Lys or His suggests that the Asn108/Gly replacement should lower the energy barrier of the rate determining step, possibly leading to a catalytically more efficient Ldh. In light of these computational results, we produced a mutated *L. planctarum *Ldh where the aminoacid Asp94, (corresponding to Asn108 of the *S. acanthias *enzyme), is replaced by Gly.

Initially we cloned the new *LDH *gene into an integrative expression vector under the control of the constitutive *Sc*TPI promoter (Table 1). The GRF18U *S. cerevisiae *strain has been transformed. Lactate production from the new Ldh and the wild-type *L. plantarum *Ldh (*i.e.*, the control) enzymes were compared, Figure 1A, B. The yeast clones obtained after transformation with the new *LDH *gene yielded a much higher lactate accumulation (11300 against 6150 mg l^-1^) and productivity (240 mg l^-1 ^h^-1 ^against 90 mg l^-1 ^h^-1 ^as determined during the exponential growth phase).

Additionally, comparable results have been obtained transforming the host cells with two identical centromeric plasmids bearing the wild-type (*i.e.*, control) or the mutated *LDH *gene under the control of the same *Sc*TPI promoter (data not shown).

**Figure 2:**
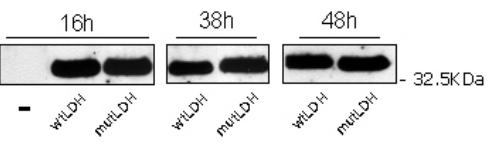
**Comparison of wild type and mutated *L. plantarum *Ldh protein levels: Western Blot determination**. *S. cerevisiae *GRF18U cells transformed with the plasmid carrying the wild type or the mutated *L. plantarum LDH *genes were shake-flask grown in glucose 2% wv^-1 ^minimal selective medium. From samples collected at indicated times total protein were TCA extracted and Ldh(s) levels were visualised by Western Blot analyses. Protein extracts corresponding to 10^7 ^cells were loaded in each lane, together with a negative control (-).

In principle, the improved production level and productivity rate could be at least associated with a better transcription rate of the mutated *LDH *gene, and/or to a lower turnover rate of the transcript, and/or to a better translation rate of the transcript and/or to a lower turnover rate of the protein. Since Ldh antibodies are not commercially available, to address these general considerations, we developed (see MM section) a polyclonal antibody against the *L. plantarum *Ldh protein. The amounts of the wild type and mutated proteins were evaluated along the growth curves of the recombinant hosts. We also determined the Ldh specific activities. On one side, Figure 2 clearly indicates that the amounts of the wild-type and mutated Ldh protein synthesized by the transformed GRF18U strain are similar. On the other side, the specific activity and the deduced Km value (calculated on crude cell extracts), of the mutated enzyme are different from those determined for the wild-type one, being 9,9 U mg^-1 ^and 1,01 mM against 3,2 U mg^-1 ^and 1,504 mM, respectively. The higher activity and the lower Km justify the higher lactate accumulation and the consequently much higher yield on glucose: 0,525 against 0,3075 grams of lactate produced per gram of glucose consumed, respectively. Surprisingly, the mutated Ldh did also lead to a similar ethanol production (Fig. 1A) and to a slightly better biomass production (Fig. 1A); these findings can be associated with a higher specific glucose consumption rate (Fig. 1B).

### Overexpression of JEN1 in yeast hosts producing different amounts of lactate

Wild type *S. cerevisiae *cells do not produce lactic acid; however, lactate can be used as carbon and energy source [22]. Lactic acid can freely diffuse through the membranes only in its undissociated form. Since yeast cells can grow on lactate even at pH values much higher than the pKA value of the organic acid (*i.e.*, 3,78), a specific transporter should be involved in the uptake of lactate. In *S. cerevisiae*, synthesis of a lactate permease takes place after transcription of the *JEN*1 gene. Transcription of *JEN*1 is induced by lactate and it is repressed by glucose [23-25]. Jen1 is the only *S. cerevisiae *member of the sialate-proton symporters subfamily belonging to the major facilitator superfamily [26].

It has been already shown that the over-expression of *JEN*1 in both *S. cerevisiae *and *Pichia pastoris *resulted in an increased activity of the lactate (inward) transport, while the deletion of *JEN*1 impaired the growth on both lactate and pyruvate [27]. A copy of *JEN*1 under the control of the constitutive GAPDH (glyceraldehyde-3-phosphate dehydrogenase) promoter has been introduced in *S. cerevisiae *cells using a centromeric plasmid; independently of the mechanisms of repression and degradation previously reported for the *JEN*1 gene and Jen1 protein, the cloning under a strong promoter allowed the constitutive expression of the *JEN*1 gene even in the presence of glucose [27].

**Figure 3:**
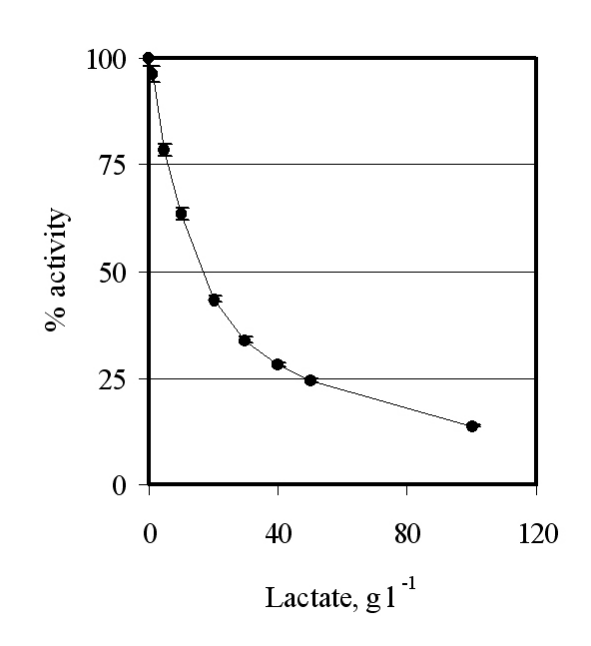
**"In vitro" inhibition of the Ldh activity by the substrate (lactate)**. Total cell proteins were extracted from GRF18U hosts cells expressing the bovine *LDH *gene. The same amount of protein extract was used to determine the Ldh activity in presence of different amounts of lactate (abscissa) and referred as percentage (ordinate) of the Ldh activity determined in absence of lactate (control).

Theoretically, higher lactate productions could be obtained by facilitating the lactate export. In fact, since the cytoplasmic pH value in yeast cells is much higher than the lactic acid pKa value, almost all of the lactic acid produced is in the dissociated form and has to be actively transported outside the cells. A limitation of lactic acid transport will inevitably lead to an increase in the cytoplasmic lactate concentration, inhibiting the "in vivo" Ldh activity and leading to the reduction or arrest of the lactate production. Indeed, Figure 3 proves that "in vitro" a lactate concentration as low as 10 g l^-1 ^reduces the overall Ldh activity by about 40%. It can be speculated that the Jen1 permease could transport lactate/H^+ ^across the two sides of the plasma membranes depending on their concentrations.

**Figure 4:**
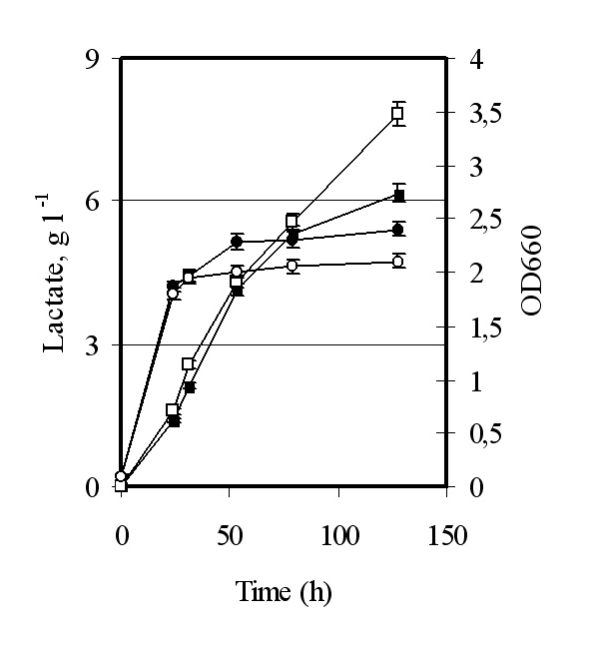
**Effect of *JEN*1 expression on lactate production in recombinant GRF18U host cells**. *S. cerevisiae *GRF18U cells transformed with the *L. plantarum LDH *(control; closed symbols) or co-transformed with the *L. plantarum LDH *and the endogenous *JEN*1 genes (open symbols) were shake-flask grown in glucose 2% wv^-1 ^minimal selective medium till stationary phase. Samples were collected at indicated times (h) for optical density (OD 660 nm, circles) and lactate (g l^-1^, squares) determinations.

Based on these considerations and looking to increase lactate export and production, we co-expressed the *JEN*1 and *LDH *genes in *S. cerevisiae *host cells growing on 2% glucose-YNB. The first findings obtained for the GRF18U yeast strain transformed with the bovine, the *L. casei *or the *L. plantarum LDH *genes showed that the co-expression of *JEN*1 was associated with an increase of both lactate production and yield. An example of such an improvement is shown in Figure 4. Results reported in the figure seem to indicate that the extracellular accumulation of the produced lactate could be modulated by the over-expression of the Jen1 permease. To test this hypothesis, we checked the lactate production from yeast host transformed with a *LDH *gene and deleted of the endogenous *JEN*1 gene. When we compared the lactate production from wild type and *jen1 *strains transformed with a *LDH *gene, no differences were observed (data not shown). Since lactate can not diffuse through the membranes, this simple observation indicates that at least another lactate transporter must be operative.

**Figure 5:**
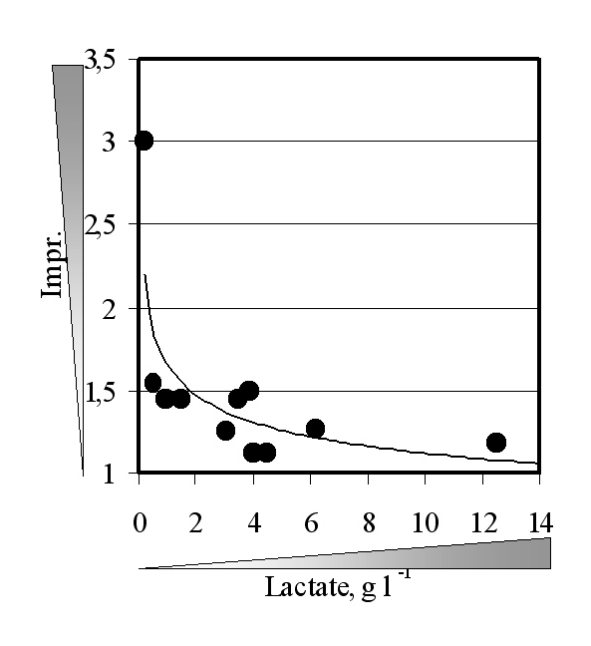
***JEN*1 effect decreases for high lactate productions**. Different yeast strains (see also Table 2) transformed with the *L. casei *or *L. plantarum LDH *genes (i.e., controls) or co-transformed with the same *LDH *and the *S. cerevisiae JEN*1 genes (see also Table 1) were shake-flask grown in glucose 2% or 5% wv^-1 ^minimal selective medium till stationary phase. In abscissa are reported the highest lactate production determined, while in the ordinate are reported the improvements observed. The improvement value represents the ratio between the highest lactate production observed in the strain co-expressing the *LDH *gene and the Jen1 permease and the same strain expressing only the *LDH *gene.

To further investigate the effect of the co-over-expression of *LDH *and of the *JEN*1 genes, we grew different recombinant yeast strains on glucose 5%-YNB. Essentially, an increase of the carbon source availability (from 2 to 5% wv^-1^) simply leads to an increased lactate production. Figure 5 reports the improvements associated to the over-expression of *JEN*1 against the total lactic acid production from different yeast strains transformed with the *L. casei *or the *L. plantarum LDH *genes. The improvement value, for any test, is the ratio between the amount of lactate produced by the yeast strain co-over-expressing *JEN*1 and *LDH *and the same strain expressing only the *LDH *gene (*i.e.*, control). A value of 1 means that an identical lactate production was observed, a value of 1.5 means a 50% higher production, a value of 2 means a double production, and so on. From the analysis of the data it can be easily evinced that co-over-expression of *JEN*1 has a clear effect when the production of lactate is very low. However, the effect of Jen1 permease becomes undetectable when the lactate production approaches about 8–10 g l^-1^, suggesting a possible saturation of the Jen1 transport mechanism.

In this respect, we did not try to further over-express the *JEN*1 gene because it has been shown that its over-expression using a multicopy plasmid did lead to an impaired growth [28].

It has been recently suggested that the export of lactate from recombinant yeast is an ATP-dependent process [10]. This extra energy required for the export of lactate could justify the lower biomass yield we generally observed for host cells producing a higher amount of lactate; Figure 4 for example compares the biomass values reached by two strains producing different amounts of lactate, being 2,4 (OD_660_) for the strain transformed with the *L. plantarum LDH *or 2,1 (OD_660_) for the same strain transformed with the same *LDH *gene and the *S. cerevisiae JEN*1.

In this respect, it is important to underline that an opposite behaviour can be obtained following the expression of the mutated *L. plantarum LDH *gene. In fact, in this case we observed a better lactic acid production/productivity and a better biomass production, being 2,4 (OD_660_) for the strain transformed with the *L. plantarum LDH *or 2,6 (OD_660_) the same host strain, but transformed with the mutated *LDH *gene. However, it should be also noted that a similar ethanol production and a higher glucose consumption rate have been obtained too. More investigations are required to better understand the biological background of this behaviour.

## Conclusion

Evolution has produced a huge variety of micro-organisms living in radically different environments. In particular, some of these micro-organisms have evolved metabolic pathways leading to the synthesis of potentially useful compounds that are difficult to produce from the chemical industry or that are environmentally harmful to manufacture. Generally speaking, the fundamental basis of evolution is the need to survive and reproduce, not to produce potentially important and commercially valuable products. Indeed, interesting metabolites are very often produced from wild type micro-organisms in such low concentrations that biotechnological exploitation is impractical. The ability to select mutants or to develop organisms by means of rDNA technologies to enable fast and environmentally friendly productions of these products has the obvious potential to revolutionize the biotechnological industry. The basic idea of metabolic engineering is to increase the flux through the selected metabolic pathway by (i) deleting (where possible) the competing branch pathways leading to the accumulation of by-products and/or by (ii) over-expressing the proteins catalysing rate-limiting steps and thereby removing bottlenecks. However, metabolic control analysis (MCA) theory teaches that, under stationary conditions, these bottlenecks do not exist. Generally speaking, all of the enzymes along a pathway are more or less equally rate-limiting [29].

As already underlined, not only for the production of important metabolites, but also for a better understanding of the fundamentals of cell biology, detailed studies are required. In this respect, we tried to modulate the lactate production and yield by improving the efficiency of the last (*i.e.*, lactate transport) and the penultimate (*i.e.*, Ldh activity) steps of the pathway leading to the accumulation of lactate from pyruvate. In fact, MCA also anticipates that a bottleneck situation sometimes could hold true for enzymes at the beginning and/or at the end of a pathway, *i.e.*, the steps controlling the starting of the pathway and the removal of the final product [29].

**Figure 6:**
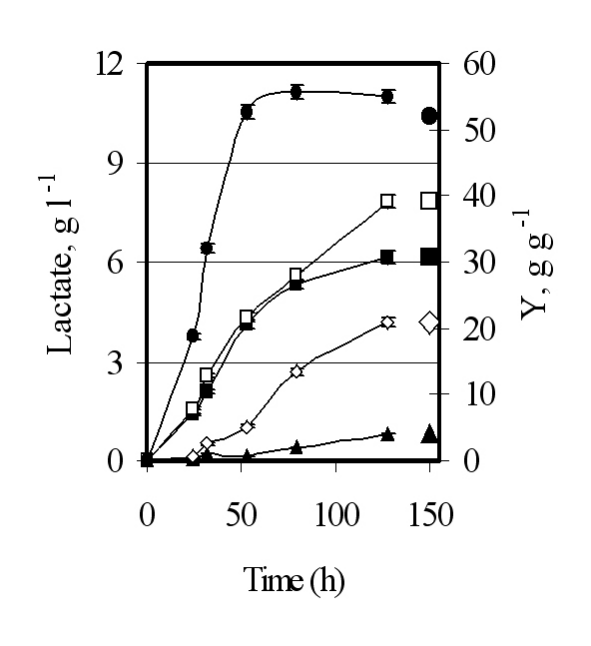
**Lactate production and yield obtained from recombinant GRF18U host cells**. Different recombinant *S. cerevisiae *GRF18U cells were shake-flask grown in glucose 2% wv^-1 ^minimal selective medium till stationary phase. Samples were collected at indicated times (h) for lactate (g l^-1^) and yield (gram of lactate produced per gram of glucose consumed; values are here below reported) determinations. () *B. taurus LDH *yield 4% ± 0.2% (☍) *L. casei LDH *yield 21% ± 0.4% (■) *L. plantarum LDH *yield 31% ± 0.5% (□) *L. plantarum LDH *+ *ScJEN*1 yield 39% ± 0.5% (●) Mutated *L. plantarum LDH*. yield 52% ± 0.7%

A yield as high as 0,52 g g^-1 ^can be reached forcing the metabolic flux towards the accumulation of the final product without any disruption and/or limitation of the other pathways competing for the same substrate. Figure 6, summarizes the production and yield values that can be achieved from the same *S. cerevisiae *strain (GRF18U) bearing different LDH growing under the same physiological conditions on 2% wv^-1 ^glucose-YNB.

Concluding, in this manuscript we proved that a high redirection of the glycolytic flux can be also obtained by modulating the last and the second last steps of the pathway leading to the extracellular accumulation of lactate from glucose. In this respect, and to the best of our knowledge, higher redirections of the glycolityc flux have never been obtained before without any disruption and/or limitation of the competing pathways.

## Methods

### Yeast strains, media and transformation

The *S. cerevisiae *strains used in this study were GRF18U [30] (MATα; *ura3*; *leu2-3,112*; *his3-11,15*; cir^+^), CENPK 113-11C (MATa *ura3-52*; *his3-11,15*;cir^+^) (see, as an example [31]), W303-1A (MATa; *ade2-1*; *ura3-1*; *leu2-3, 112*; *his3-11, 15*; *trp1-1*; *can1-100*) [32] and MB11 (MATa; *ade2-101*; *ura3-52*; *lys2-801*; *his3-Δ200*; *trp1-Δ1*; *can1*) [33].

Yeast cultures were shake-flask grown in minimal synthetic medium (0.67% wv^-1 ^YNB Biolife without amino acids) with 2% wv^-1 ^of glucose as carbon source. When required, supplements such as leucine, uracil and histidine were added to a final concentration of 50 mg l^-1^. The cultures were repeated independently (at least) three times, samples were taken at different times for the determination of growth parameters, metabolite production, nutrient consumption, enzymatic activities and protein level.

Transformation of *S. cerevisiae *strains was performed according to the LiAc/PEG/ss-DNA protocol [34]. The yeasts were transformed with one or more of the constructs described below, together with the respective empty plasmid(s). For each set of transformation, independent clones (at least 4) were initially tested and lactate levels determined, showing no significative differences among the independent clones.

### Genes amplification, mutagenesis and expression plasmid construction

For the expression of the different heterologous LDH activities and for the over-expression of the *S. cerevisiae *lactate transporter Jen1, yeast expression plasmids of the series named "pYX" (R&D Systems Wiesbaden, Germany) have been used. In these plasmids, the cassette for heterologous protein expression is based on the constitutive *S. cerevisiae *TPI promoter and the respective polyA, interspaced by the multi-cloning site (MCS), exploited for the sub-cloning of the genes of interest. The selection is based on different *S. cerevisiae *auxotrophic markers (in the present study always indicated in parenthesis, see also Table 1). In particular, the series of plasmids here utilised are all integrative, and the efficiency of integration was optimised by linearizing the plasmids before the yeast transformation, exploiting a unique site present in the sequence of the auxotrophic marker.

The full length *Bos taurus *(bovine) LDH-A coding sequence was PCR amplified from the plasmid pLDH12 [35] and inserted in the pALTER-1 (Promega) vector as already described [36]. From the resulting plasmid pVC1 the bovine *LDH *was sub-cloned into the commercially available yeast integrative vector pYX012 (*URA3 *marker) generating the expression plasmid pB1. Alternatively, the bovine *LDH *gene was sub-cloned into the integrative plasmid pYX022 (*HIS3 *marker) and pYX042 (*LEU2 *marker), generating the plasmids pB2 and pB3, respectively. For the described expression plasmids, the enzyme coding sequences were all *EcoR*I/*Sal*I excised, and sub-cloned in the receiving expression vectors equally opened.

The *Bacillus megaterium *and *Bacillus stearothermophylus LDH *genes (sequences available at the accession n. M22305 and M19396 GenBAnk, respectively) were PCR amplified from genomic DNA of the respective strains obtained by using the Invisorb Spin Bacteria DNA Mini Kit (Invitek), following manufacturer's instructions. The following primers were designed and used: *LDHBM fw*: 5' ACA AAT GAA AAC ACA ATT TAC ACC AAA AAC 3' *LDHBM rev*: 5' ATG TTA CAC AAA AGC TCT GTC GC 3'; *LDHBSt fw*: 5' AAT GAA AAA CAA CGG TGG AGC CCG 3' *LDHBSt rev*: 5' tgc ctc atc gcg taa aag cac gg 3'. The respective unique fragments obtained were sub-cloned in the vector pSTblue-1 utilising the Perfectly Blunt^® ^Cloning Kit (Novagene) and checked by sequencing analysis. From the obtained plasmids pSTBMLDH and pSTBSTLDH, the coding sequences were *EcoR*I excided and subsequently sub-cloned into the *S. cerevisiae *integrative expression vector pYX012 (*URA3 *marker), *EcoR*I opened and de-phosphorylated. The resulting expression plasmids were named: pBME2 and pBST2, respectively.

The *Lactobacillus casei LDH *gene was PCR amplified and subsequently sub-cloned into the yeast integrative vector pYX012 (*URA3 *marker) generating the expression plasmid pLC5, or in pYX022 (*HIS*3 marker) generating the expression plasmid pLC7, as previously described [30].

The *Lactobacillus plantarum LDH *gene (sequence available at the accession n. X70926, GenBAnk) was PCR amplified from genomic DNA obtained from the strain ATCC 8014 by using the Invisorb Spin Bacteria DNA Mini Kit (Invitek), following manufacturer's instructions. The following primers were designed and used: *LDH fw*: 5' TGA CTT ATT ATG TCA AGC AT 3', where a mismatch was introduced in order to substitute the bacterial start codon TTG with ATG, correctly recognised by *S. cerevisiae*, and *LDH rev*: 5' ATC GTA TGA AAT GAT TAT TTA TT 3'. The unique fragment obtained was sub-cloned in the vector pSTblue-1 utilising the Perfectly Blunt^® ^Cloning Kit (Novagene) and sequenced. In the amplified sequence four bp resulted substituted in respect to the original sequence, but only two of them generated two conservative aa substitutions (H54D and C302S, respectively). From the obtained and sequenced plasmid pSTplLDH, the coding sequence of *plLDH *was *EcoR*I excised and subsequently sub-cloned into the *S. cerevisiae *integrative expression vector pYX022 (*HIS3 *marker), pYX012 (*URA3 *marker) *EcoR*I opened and de-phosphorylated. The resulting expression plasmids were named p022TLP and p012TLP, respectively.

The *plLDH *was then also mutagenised according to the suggestions derived from modelling studies (see below). In particular, the aa 94 Asp (D) was changed into Gly (G) by a double bp substitution changing the triplet GAC into GGT. The mutagenesis was made by the Altered Sites II in vitro Mutagenesis Systems (Promega) using plasmid pSTplLDH as template DNA and complementary primer pairs that encode the desired amino acid replacement, following manufacturer's instructions. The sequence change was confirmed by DNA sequence analysis and finally sub-cloned into plasmids of the already described series "pYX". In particular, the modified sequence was inserted in the plasmid pYX022 (*HIS3 *marker) or in the plasmid pYX012 (*URA3 *marker), resulting in the yeast expression plasmids p022TLPD94G and p012TLPD94G, respectively.

The DNA sequence of *JEN*1 (available at the accession n. U24155, GenBank) was PCR amplified with the following primers: Tony s: 5' ACT GCT ACT GAA AAT ATG TCG TCG T 3' and Tony as: 5' AGT GAT TAA ACG GTC TCA ATA TGC TC 3' starting from *S. cerevisiae *genomic DNA extracted as described (slightly modified from [37]). The unique fragment obtained was sub-cloned in the vector pSTblue-1 and sequenced. From the obtained plasmid, the coding sequence of *ScJEN*1 was *EcoR*I excided and subsequently sub-cloned into the *S. cerevisiae *integrative expression vectors pYX012 (*URA3 *marker) and pYX022 (*HIS3 *marker) *EcoR*I opened and de-phosphorylated, generating the plasmids p012Jen1 and p022Jen1, respectively.

DNA manipulations, transformation and cultivation of *Escherichia coli *(DH5αF' (φ 80d *lacZ*ΔM15, Δ (*lacZYA-argF*), *U169*, *deo*, *rec1*, *end1*, *sup44*, λ, *THI-1*, *gyrA96*, *relA1 *and Novablue Competent Cells (Novagene) were performed following standard protocols [38]. All the restriction and modification enzymes used were from New England Biolabs (Hitchin, Herts, UK) or from Roche Diagnostics (Mannheim, Germany).

### Measurement of cell concentration, metabolites and enzymatic activities

Independent recombinant yeast transformants were shake-flasks cultured in minimal medium. During the cultures, followed up to stationary phase, samples were collected at regular time intervals. Cell concentration was determined by measuring the optical density at 660 nm or the cell number by a Coulter Counter determination [39].

Glucose, ethanol, L(+)-lactate were determined by using diagnostic kits (Boehringer Mannheim cat n° 148261, 716251 and 176290, respectively) according to manufacturer's instructions. The lactate yields were calculated by linear regressions obtained by plotting the grams of glucose consumed in the course of fermentation processes *versus *the grams of lactate produced.

For all LDH activities, samples were prepared as follows: about 10^8 ^cells were harvested, washed in ice-cold water and re-suspended in 50 mM phosphate buffer pH 7.5, Glycerol 20%, PMSF 1 mM and protease inhibitors (Complete EDTA-free Protease inhibitor cocktail tablets, Roche). Cells were broken with 5 cycles of vigorous vortexing in presence of glass beads (diam. 400–600 μm, Sigma) at 4°C. After centrifugation, protein extracts concentration was determined (Bradford, Biorad).

For bovine LDH activity, about 0.2 mg of extract were tested using the Sigma kit DG1340-UV, according to manufacturer's instructions.

For all bacterial LDH activities determination, except the *L. plantarum *one, cellular extract (0.05 ml of properly diluted samples) were incubated with 0.01 ml of 12.8 mM NADH, 0.1 ml of 2 mM fructose1,6-diphosphate, 0.74 ml of 50 mM acetate buffer (pH 5.6) and 0.1 ml sodium pyruvate 100 mM. The *L. plantarum *LDH activity was almost identically determined, omitting the not necessary cofactor fructose1,6-diphosphate, as previously described [40]. LDH activity was assayed as micromoles of NADH oxidized per min, per mg of total protein extract, at 340 nm, 25°C.

### Simulations

The computational investigation has been carried out using the X-ray structure of Ldh from *Squalus acanthias*, chosen because both of the holo and apo structures are available (pdb codes: 3LDH, 6LDH, respectively. Web site: ). However, results are expected to be valid also for Ldh from *L. planctarum*, due to the high sequence similarity between the two proteins (not shown).

Molecular Mechanics (MM) optimizations were performed using the GROMACS simulation software package [41], implemented on a parallel architecture. MM runs consisted in 1000 steepest descent cycles followed by 10000 conjugate gradient steps.

Initially, cofactor (NADH) and substrate (pyruvate) have been removed from the X-ray structure, in order to simplify the computational investigation of the conformational properties of the protein. Since the movement of the catalytic loop takes place on a relatively low time scale (kcat = 250 s^-1^), average value among several species [20] standard Molecular Dynamics simulations could not be used to investigate the conformational rearrangement. Instead, the conformational properties of the catalytic loop have been investigated by iterative modification of selected torsional angles, followed by Molecular Mechanics optimization. In particular, analysis of the X-ray structures of the apo and holo form of Ldh suggests that the conformational rearrangement of the catalytic loop mainly implies modification of two protein torsion angles (phi_96 _and phi_97_, N-C_alfa_), which have been systematically rotated in steps of 10 degrees. The resulting structures were optimised by MM in order to obtain stationary points on the Potential Energy Surface (PES), thus allowing to sample the PES of the protein structure along the reaction coordinate corresponding to the relevant movement of the catalytic loop.

### Antibody development against L. plantarum LDH and protein level analysis

Different commercially available antibodies against LDH were tested, but none of them is specific for bacterial LDHs and none of them resulted sufficiently specific for *pl*LDH detection in our host system. By utilizing the tool Syfpeithi  we individuated the peptide DCKDADLVVITAGAPQKPGE (from aa 71 to aa 90 of the aminoacidic sequence of the protein) as the preferred immunogen to develop polyclonal antibody. The above mentioned peptide and the corresponding antibody (as immune serum) were synthesized and developed, respectively, by Primm Italia .

LDH expression levels were analyzed by Western Blot. From each point of the above described kinetics of growth, a culture volume corresponding to 10^8 ^cells was harvested by centrifugation, and crude extracts were prepared by following the trichloroacetic acid protocol [42] and resuspending the final protein extract in 150 μl of Laemmli buffer [43]. Protein extracts corresponding to 10^7 ^cells were loaded on a 12% polyacrylamide gel (SDS-PAGE); after separation, proteins were blotted to nitrocellulose membranes and immunodecorated. LDH levels were detected using the developed primary rabbit polyclonal antibody, (dilution 1:250) and a secondary anti-rabbit IgG horseradish peroxidase-conjugated (Amersham Pharmacia Biotech, UK; dilution 1:5000) antibody. The immunoreactive protein bands were developed using the Super Signal West Pico Western blotting system (Pierce), according to the manufacturer's instructions.

## Authors' contributions

PB participated in the design of the study and planned and carried out the experimental work including plasmid and strain construction, yeast cultivation and data analysis, western blot immunoassays and enzymatic activity determination; participated in drafting the manuscript.

MS and MV participated in the design of the study and in molecular biology experiments.

LDG and GZ designed and performed the modeling studies and simulations, suggesting the point mutation.

DM participated in the design of this study and in data interpretation.

DP designed the whole study, performed data interpretation and drafted the manuscript.

All authors read and approved the final manuscript.
